# Atlanta Academy of Medicine

**Published:** 1876-08

**Authors:** J. S. Todd

**Affiliations:** Reporter


### Atlanta Academy of Medicine.

J. S. TODD, M.D., REPORTER.

ATLANTA, June 13, 1876.

The Vice-President, Dr. John G. Westmoreland, presiding.

Dr. R. W. Westmoreland called attention to a case of premature delivery of dead fœtus at sixth month. The mother was first seen by him a month prior to the abortion. She was then flooding. She had menstruated regularly, though profusely at each time, up to the misshap. Her condition was not suspected by any of the physicians who saw her. The terrible odor of the discharge, two or three days prior to the occurrence of the accident, was the first symptom that caused attention to be turned in this direction. Remarked that it was an unusual case, because of the regular monthly discharge during gestation.

Dr. Todd said he had nothing to say as to the cures wrought by Drs. Miller and Johnson with ammo-murias. They were scientific men, close observers, and of veracity unimpeached. Dr. Miller, however, stated for fact, that which recent research had controverted, namely: The action of ammonia on fibrin, and its power of preventing blood from coagulating. This foundation of the therapeutic action of ammonia was built on a theory long since undermined, as will appear from the following quotations from 1st Brand & Taylor's Chemistry, edition 1867, article Fibrin: "It has been shown by Dr. Davy and others, that healthy blood contains no appreciable quantity of ammonia, and that ammonia added to fresh blood in any quantity did not prevent the fibrin from assuming the solid state.—[Ed. *Journal*, 1859, vol. xlv., page 537.]

When ammonia has been found in blood it has been probably the result of incipient decomposition.

It is also a curious fact that ammonia is the only one of the common alkalies which has no solvent action on fibrin when it has once solidified. This being so exactly contrary to Dr. Miller's theory, he deemed other evidence necessary, and accordingly consulted the largest and best work on chemistry extant, viz: and secondly, a Dictionary of Chemistry, in six volumes, of about 1900 pages each, by Henry Watts. He says, article Fibrin, head 3: "Fibrin is scarcely acted upon by ammonia."—Edition of 1872.

3d. Fownes' chemistry, dated 1876: "Fibrin is soluble in concentrated acids and alkalies and in ammonia-cupric solutions, but not ammonia."

4th. Dictionary of Chemical Solubilities, by F. H. Storer: "Fibrin is soluble in even very dilute solutions of caustic potash, but not ammonia water."

Dr. Richardson published his supposed discovery, which was essentially, as stated by Dr. Miller, in 1858, and received for it the Astley Cooper prize. Davy and others, in the year following, showed that his experiments were wrong. Hence, the comparatively little reputation of ammonia as a remedy in inflammatory disorders, and as a popular curative agent in disease.

Dr. Taliaferro said, in recalling the discussion of last meeting, granting that ammo-murias did dissolve fibrin, and that inflammatory deposits were taken up by the blood during its administration, that he did not see how it could cause the absorption of a fibroid of the uterus; for such tumors were not the result of inflammation, or caused by fibrinous deposition. A fibroid was a tumor, homologous in its structure, whose tissues were identical with those of the uterus. And again, their extreme difficulty of diagnosis was so noted, that on several occasions the most learned and eminent in our profession had been so misled as to actually amputate the inverted uterus, mistaking it for fibroid, and opened the abdomen for the removal of tumors where none existed. Therefore, with all due deference to Drs. Miller and Johnson, they would pardon him if he was unwilling to hastily conclude that they always had a fibroid to deal with when they so thought. In enlargement of the uterus from sub-involution—in chronic inflammation causing an increase in the bulk of that organ—modern pathology did not refer the increase in size in the one instance, and the failure in the other to resume its natural dimensions, to fibrinous exudation. The increase in size is generally in the cellular and muscular tissues. It is not claimed that the remedy will act on those tissues. If the remedy does what is claimed for it, to keep a person on it for several months, would cause the opening of old cicatrices, and no wound would heal, with the blood in the aplastic condition which ammo. induces, granting the theory advocated by Dr. Miller correct.

Dr. Baird did not agree with Dr. Taliaferro in reference to the histology of fibroids. The typical fibroma in its textural elements resembles cicatricial tissue, and consists of a very dense fibrous intercellular substance, and small oval cells with shining nuclei, besides rows of spindle-cells that traverse the tumor in different directions. He had nothing to say as to the power of the muriate of ammonia in causing absorption of these tumors, but the explanation offered at the last meeting, viz: its power to dissolve and absorb fibrin is not applicable. These tumors cannot be regarded as deposits of fibrin from which the defibrinated blood may be supplied, for as has been shown, they are not composed of fibrin at all.

The same objection holds to the explanation offered of its alleged resolvent action on goiter. This tumor is not composed of the thyroid body distended with fibrin, but it is a true hyperplasia of the glandular substance, and whatever form it may subsequently assume, whether cystic, vascular, gelatinous, fallicular or amyloid, hyperplasia of the normal structural elements is always the first step. The thyroid body is composed of glandular follicles, consisting of closed vesicles, containing a drop of clear albuminous fluid, a frame-work of connective tissue and blood-vessels. In hypertrophy, the growth of any of these elements may predominate. We may have an increase mainly of the glandular substance, or of the connective tissue, or an abnormal increase of the vascular element. In the first form, as the growth progresses, the septa of adjacent cells may be absorbed, and a common cavity of many cells result, forming a multilocular or sometimes a unilocular cyst, with very thick walls, the lining membrane then becomes a secreting membrane, and the distention is still further augmented by the accumulating fluid. The contents of these cysts may be colloid in consistency, representing still another variety—or the gland may be infiltrated with calcareous or amyloid material. The facts presented by Dr. Miller are not questioned, but only his explanation of the process by which a cure is accomplished. So far as muscular hypertrophy is concerned, whether affecting the pregnant uterus, the heart, or voluntary muscles, he is aware that it is generally thought to be due to increase in size of the individual fibers and not an increase in their number. The impossibility of estimating the number of fibers in any muscle is manifest, and while the growth is undoubtedly due in part to increased size of the fibers, it seems to him that it is reasonable to refer it, in part, to an actual addition of contractile cylinders. Take the biceps of a blacksmith. The individual fibers may be larger than those of a child, but is it not probable that there are more of them? Suppose that the requisite number of fibers existed in childhood, did they exist six months before birth? They certainly did not nine months before. The question then is at what age does the capacity for the new formation of the muscular tissue cease? He thought the same rules pertaining to the growth of other tissues applicable to muscular tissue, that the elements of muscular growth resided in the muscle, and that under the influence of certain stimuli, the muscle increased both in size and number of its fibers.

Dr. Taliaferro said, the difference between his ideas and

Dr. Baird's—which were histiologically correct—was more in words than effect, and arose from the nosology of these tumors being in a manner unsettled. Hence the terms were often confused and fused. Myoma, fibroma, and by others myo-fibroma, were by others all classed as fibroids. As to there being new muscular tissue in the uterus during pregnancy, he was aware that the question was *sub-judice.* He believed there was both hypertrophy and hypergenesis.

---

ATLANTA, June 20, 1876.

H. V. M. Miller, A.M., M.D., L.L.D., presiding.

Dr. Taliaferro said, recently his views as to the propriety of giving quinine in pretty large doses, in typhoid fever, had undergone a change. Formerly in treating it he had not been in the habit of administering this drug, but he had lately given it in conjunction with opium, and was much pleased with its effects in diminishing the pulse and fever; quieting nervousness and controlling the bowels. The opium of course had much to do with allaying the last two. He pays, if possible, more attention than ever to nourishing his patient. He also allows whisky or brandy *re nata.* The condensed milk, a drachm to half a gill of warm water, and barley or oat meal tea are his favorite aliments. He gives the quinine every six hours in five grain doses, with half a grain of opium. Under this treatment he is satisfied that patients do not loose so much flesh. He treated a case recently, a young man sick five weeks, who weighed the third day after being up, and his avoirdupois was actually, so he said, three pounds greater than before the attack. Another case, now under treatment, and has been for three weeks, has not lost flesh perceptably. In one case he supposed the oat meal tea aggravated the diarrhœa.

Dr. J. G. Westmoreland asked what benefit did he hope to accrue from quinine?

Dr. Taliaferro—To lessen fever, control circulation and for its anti-septic influence. He believed the etiology of the disease might, and could be, traced to certain substances, atmospheric, which caused putridity of the blood.

Dr. J. G. Westmoreland was opposed to quinine, because he thought it was an irritant to the alimentary mucous membrane, and would tend to increase diarrhœa, and cause serious lesions in Pyers' glands. Unless it prevented fever, or cured the disease, he would not give it. He has in persistent malarial fever, where quinine was given, caused manifest and troublesome intestinal irritation. He thought the good to be expected counterbalanced by this action of the remedy on the stomach and bowels.

Dr. Taliaferro said he once thought as did Dr. J. G. Westmoreland, but was now satisfied that it did not act as an irritant, unless it was immediately applied; that is, brought in direct contact. The opium causes it to be tolerated. Where it failed to do so he would give hypodermically. He mentioned a case, as illustrative of its tolerance with opium, where it was rejected when administered alone. If it was an irritant, other than local, to the alimentary mucous membrane, that effect would not be obviated by hypodermic injections, as he knew, by experience, was the case.

Dr. J. G. Westmoreland said he has now a case under observation which has been taking large doses of quinine, under the supposition that it was a malarious fever. To-day diarrhœa symptoms are present which he attributes, in a large measure, to the quinine.

Dr. Baird to Dr. Taliaferro—Do the doses of quinine you give cause tinitus aurum?

Dr. Taliaferro—Yes, a little hardness of hearing in one case.

Dr. Baird—Is there nervous twitching of the muscle—of the wrist, for instance?

Dr. Taliaferro—Some sub-sultus, but not troublesome.

Dr. Baird thought twenty-grain doses depressant. The Germans gave it in continued fever, but stopped its use after three or four days and then resumed, thus alternating.

Dr. Taliaferro—He gave it as a nervous stimulant, and as such regard it as one decidedly. He attributes his patients not loosing flesh to its bracing effect.

Dr. Baird—In large doses it is not a stimulant or tonic. For these effects we should stop short of tinitus aurum, and asked Dr. Taliaferro what would be his guide as to how much should be given or when enough had been taken? As the flushed face indicated too much alcohol, so did tinitus aurum proclaim the depression of cinchona alkaloids.

Dr. Taliaferro—We must give with judgment. In nervous depression larger doses of alcohol were necessary than in health. The poison of typhoid fever acted as a fell paralyzer of the nervous system, and large quantities of alcohol and quinine were required before the depression caused by these remedies was induced.

Dr. J. G. Westmoreland—That which irritates excessively, finally depresses. For instance, opium.

Dr. Taliaferro—Persons in malarious regions take twenty grains, and even forty grains at a dose, pursuing their ordinary avocations. Depression does not result in these instances.

Dr. Baird—In those instances it is neutralized by the malaria. Same doses in health caused great nervous depression; the annoyance was excessive; the feeling of "giving out" conspicuous. Small doses were tonic. Appealed to Dr. Miller for his experience on this point.

Dr. Miller—In his own case, Dr. Baird was correct. Had no experience with it in typhoid fever. A large dose depressed him exceedingly.

Dr. J. G. Westmoreland—In small doses quinine is not a tonic. He knows of no antiperiodic property that it may possess. When he gives it, if no tinitus is produced, does not think he has done any good.

Dr. W. F. Westmoreland—The question is, can we cure typhoid fever. He had as soon try to cut short measles, small-pox, or any disease which had a certain course to run. Of this class, he considered typhoid fever a member. He only treats the symptoms as they arise. Doctors kill their hundreds: yes, their thousands, by physicing and seeking to cure that which, left to nature, would exhaust itself. He only interferes with the accidents; he does not attempt to direct it. He, of course, believes in nourishment, but uncomplicated cases will get well if left alone and fed.

Dr. Taliaferro--I do not claim to cut short an attack, or cure the disease, but I do propose to lessen the pulse, prevent the waste, etc. I hold that when I am giving a nervous tonic *I am* helping nature. We must sustain the powers of life, or death will result. Even uncomplicated cases, in the majority of instances, without attention other than mere feeding, would die. Does not regard small-pox and fever as lightly as Dr. W. Believes in the vis medicatrix naturæ as strongly as any one, but at the same time will not leave all to her unaided powers.

Dr. Miller briefly reviewed the history of cinchona and its alkaloid, quiniæ. When cinchona first came to be used, it was fashionable to give small doses—never during fever; so with quinine; hence they often failed, and the lancet, mercury, etc., were used instead. Later large doses, and it was a demonstrated fact that quinine would interrupt and cure the paroxysms of intermittent and remittent fevers. Some—the majority—called it an antiperiodic, which was a term which expressed our ignorance. How it cured was not known. It is generally supposed that cinchona is a tonic in small doses; the failure of small quantities to effect cures, shows that it does not cure because of being a tonic. Where typhoid fever has worn the livery of malaria it has been used with success. It has been a general practice to give quinine in small doses as a tonic during continued fever. The Germans give 15 grains at a dose—three doses in rapid succession at the height of the fever, and use the cold bath. They claim that it lessens the temperature and pulse. If it will more certainly do so than veratrum, then he advises it, but has no personal experience to confirm their observation. Observed that a reduction of temperature and pulse was the very opposite of a tonic effect. That it is antiseptic and neutralizes or eliminates certain septic materials from the blood, he thinks it should first be shown that such have an existence in that fluid. If the disease depends on a poison which quinine neutralizes, then it should cut short an attack. He considered nutrition absolute in treating the disease; also used veratrum and cold sponging to control pulse, and thereby heat. Dr. Forte, of Milledgeville, relied exclusively on opium. He could not see the philosophy of giving alcoholic stimuli, when your desire was to control the action of the heart. Some claimed immense success by treating with the lancet.

Dr. Taliaferro—Alcohol and quinine do not increase the temperature. He has also given veratrum, when the quinine and opium were not of themselves sufficient. Has also observed that when the three are given together, the veratrum must be in smaller dose than when given alone, to obtain its sedative effects.

Dr. Baird repeated his opinion, that small doses were tonic, large, depressant; therefore they were contra-indicated in typhoid, continuously administered.

Dr. J. G. Westmoreland—When he gave a large dose of quinine to interrupt a paroxysm in remittent, or a chill in intermittent, and it failed, he always noticed that the fever which followed was higher.

Dr. Connally's experience was not uniform. He had, he was certain, in malarious fevers given large doses of quinine, and thereby caused his patient to break out in a perspiration, etc.; but again he had, he thought, caused the fever to assume a higher grade. On the whole he is rather inclined to favor large doses—20 grains to be given to cause a reduction of pulse and temperature.

Dr. Taliaferro had never, in treating remittent, hesitated on account of the fever to begin quinine, and has every reason to be pleased with his practice.

---

ATLANTA, June 27, 1876.

The President, Dr. Miller in the chair.

Dr. Calhoun exhibited specimen of tumor extracted from orbit of eye. It was quite large to occupy that cavity. He had not examined specimen with microscope, but thought from history and appearance that it was a cancerous (sarcornatous) growth. It was not clear from the history of family that it was of a cancerous nature, as no member of his family, except an uncle probably, had suffered from that disease. It had grown slowly from its first appearance three years ago, owing to resistance from the bony walls of orbit. Until within a short time its augmentation had been rapid, since it had been freed from the resistance of the ball below and bones above. Its weight was estimated at four ounces. The patient's age is forty-five and his health is good. Has so far given no pain. It caused complete dislocation of eye ball, giving the appearance of exopthalmic goitre. The ball was forced downwards and forwards so that the pupil was three-fourths of an inch lower, and the anterior surface of the cornea one inch in advance of the same points in the other eye. The tumor filled the entire cavity of orbit, paralyzing upper lid and precluding all upward motion of ball. Could count fingers at only three feet. Ophthalmoscope revealed congestion of retina and hemorrhage spot upon the face of the nerve.

The operation for its removal was performed under the influence of ether and chloroform in combination. An incision was made on outside of upper lid one and a half inches in length, from external canthus to the internal, along the supra orbital ridge. It had to be dissected from bony orbit, which caused much difficulty in reaching posterior attachment which was around optic foramen, more or less completely grasping the nerve.

He had to cut away a portion of the growth before he reached its origin, in order to get at the posterior part of tumor. At one point the roof of the orbit had been absorbed, so great was the pressure, leaving a perforation that he could lay his finger in. The optic nerve from the foramen to ball was about one and a half inches in length. It resumed its original length soon after operation. There has been rapid healing despite the rough handling. The pupil of this eye, which was three-fourths of an inch lower than corresponding pupil of other eye, is now on a level with it. He left a tent in wound, from which there is a slight discharge still. Vision, ¼ normal, can read No. 40 at ten feet. The paralysis of superior rectus and lid owing to the long pressure is likely to remain.

Dr. Rauschenberg said he was called to see a case on Monday last, the man had been suffering from his bowels since the Wednesday previous. Found them typanitic and constipated. He was the subject of an old hernia of many years duration; its character being inguinal of right side. There was a tender spot immediately above ring. The hernia, which had been freely reducible heretofore, he found impossible to get back. He bled him and still failed to reduce. Gave in the evening one grain morphine, an hour later, half a grain more, which, in an hour, was repeated, and he again endeavored to replace the intestine but failed. Next day sent for Dr. W. F. Westmoreland in consultation. As the man's bowels were freely opened by and responded to purgatives, they waited for two days longer, meanwhile the irreducible tumor continued to increase and exhibited slight fluctuation. Sunday an opening with bistoury let out a pint of pus. Tuesday a teacupful was discharged, and Wednesday half an ounce. There was much fœtid gas also escaped.

Dr. W. F. Westmoreland thinks it not clearly demonstrated what the disease is. Doubts its being a hernia, and thinks it probable that the ring was filled by omentum. Is inclined to think it perityphlitis. The horrid fetor of discharge made him sick; for anything to cause that phenomenon in him must be awful indeed. This character of discharge could only be generated in the intestine. Showed how nature would throw around a wall of lymph, and prevent contents of bowels from emptying into peritoneal cavity. The tenderness spoken of by Dr. R. was not in the portion of mass external to ring, but just above it. The tumefaction and induration covered a space on abdomen 4 by 6 inches. He punctured one-half an inch above the ring. He rather doubts there being any intestine in it, however.

Dr. J. T. Johnson said similar cases were often reported in the journals, and called perityphlitis, where a wall was formed and protected the peritoneal cavity. He had had a case that he was sure died with it, but was not permitted an examination. The case alluded to was complicated with typhoid fever.

Dr. J. M. Johnson once treated a gentleman 50 years of age, who had a tumor in right iliac fossa. Nevertheless he continued to plough for two weeks, then went to shoemaking, because of the difficulty of locomotion; finally had to take his bed. After a course of poultices, the swelling was lanced, which discharged an immense amount of pus, and a *cherry stone.* This, or some foreign body, may cause the mischief in Dr. R.'s case; or the irritability being a recent feature, may be caused by excessive irritation of ring, etc.

Dr. J. M. Johnson reported a case of typhoid fever. On the 5th of June he was called to see Miss W., aged 19, unmarried. She is a citizen of Macon, Georgia, but on a visit to some relatives in this city. She had considerable fever, and greatly disturbed bowels, and as dysentery was prevalent he prescribed castor oil and laudanum, followed by quinine and dovers powder. The oil acted well, indeed rather rashly, notwithstanding the laudanum. The quinine and dovers powder was given, with relief to the bowels, but the fever continued. He was summoned next day to see her again. Found the bowels at rest, but the fever much greater, and attended with rigors. Upon counting the pulse on this day (6th June) he found it to be 120 beats per minute. He then gave Norwood's tinct. veratrum, and calomel and dovers powder. On the 7th found pulse down to 90 and skin soft; gave castor oil, to be again followed by quinine and dovers powder. On the 8th, found that the castor oil had again acted rashly, but the purging was controlled by the dovers powder. The pulse 110, although the tinct. veratrum had been persistently given, even to nausea, which he directed should be suspended if nausea occurred. The tongue, which had before presented no indications of irritation, was covered with a brown fur and reddened tip and edges. It had also contracted to a wedge. He saw at once that he was dealing with typhoid fever. As she had suffered a good deal with pain in the back and hips, and also with cold feet from the beginning, he had ordered warm hip and foot baths, which had been taken from three to five times daily, and always with benefit. On the 8th June, at night, he put her upon an emulsion of turpentine, camphor, and elixir of opium, as follows: turpentine eight drops, spts. camphor eight drops, elixir opium four drops every four hours, and directed the surface of the abdomen rubbed with spts. turpentine, and grease enough to keep it from irritating the skin, also every four hours, and an enema of black drop (twenty drops in a teaspoonful of warm water) whenever the bowels threatened to act. On the 9th at 7 o'clock, found the patient resting well, pulse 110, tongue moist, broader, and less red. She had one bath during the night. Ordered chicken essence, or milk, or both, in small quantities every four to six hours—also a little brandy in all the water she drank—and continued the remedies, with directions to give veratrum as before. Saw patient at 8 P. M., pulse 120; no other visible change. Had taken food freely, but without appetite, except for butter-milk, of which she had taken a pint; complained of the brandy; had two baths and enjoyed them very much. Treatment continued. June 10th, 7 o'clock A.M., pulse 102, had rested well, skin moist, had no bath during the night, took food twice, and brandy in all the water she drank. Treatment same. 10th, 8 o'clock P.M., pulse 108, tongue cleaned off, redness less, had taken food with some pleasure; begged for wine instead of brandy, which was allowed; one bath taken. June 11th, 7 o'clock A.M., pulse 96, had a good night, says she wanted coffee instead of chicken essence; complained of uneasiness in her bowels, and desires to have an action. Ordered an enema of warm water, which was followed by a discharge of healthy matter, but gave black drop to lock up and quiet everything. Treatment continued. 8 P.M., pulse 100, had two baths, had taken milk freely, felt stronger. June 12th, 7 A.M., pulse 110, some muttering during sleep, and twitching of the muscles; complained of general malaise. Upon examination found some tympanitis of bowels. Ordered an enema of very warm soap-suds, sufficient to fill the colon—say more than a gallon—followed by a large action and an abundant discharge of gases. After this action was had, he ordered an enema of black drop (thirty drops to two teaspoonfuls of hot water), after which she slept well for four hours, and awoke refreshed and happy. 8 o'clock P.M., pulse 102, skin good, had taken chicken essence and milk freely, and asked for more than the allowance. 13th, 7 o'clock, tongue almost natural, pulse 90, wants to know if she can set up. 8 P.M., pulse 100, has not eaten so much, not so strong, don't feel able to sit up. Resumed the brandy—bad feelings due to over-eating. 14th, 9 o'clock A.M., pulse 85, feels well. 8 P.M., pulse 88. Case dismissed on the 21st day after the attack. It will be seen that quinine was freely given for three days without the least apparent benefit at the beginning of the treatment, and none was given after the real character of the fever was ascertained. The rigors only disappeared after repeated hot bathings, and the bowels were thoroughly checked and soothed by opiates. He attributes the cure to this, together with the careful nursing and feeding from very devoted friends.

NOTE.—Dr. Todd asked if there was an eruption. Dr. Johnson replied he did not know, but upon questioning the patient the day after report, she stated to him that a flea-bitten eruption was thickly broken out on her body, inside of arms and legs.

